# Pragmatic constraint-led approach to sample size

**DOI:** 10.1186/1745-6215-16-S2-P27

**Published:** 2015-11-16

**Authors:** Paul Silcocks, Diane Whitham

**Affiliations:** 1 University of Liverpool, Liverpool, UK; 2 University of Nottingham, Nottingham, UK

## 

Standard approaches to sample size specify power, significance, treatment effect, one-or two-sided test and allocation ratio, to yield the number of patients. Typical reactions are “I can only get 60”. Statisticians often respond by seeing what effect the available number can “buy” for reasonable significance/power.

This approach can extend to other constraints. Time is the most important because “How quickly we need the answer” defines the overall duration, partitioned into setup & closedown, minimum follow-up (enough to see response), and accrual time (related to number of sites, rate of site opening and recruitment per site). The last site opened must also contribute patients before accrual ends. From these quantities the number of patients follows, but will this be enough to detect a signal?

A signal is strong evidence favouring H1 rather than H0, encapsulated in the likelihood ratio (LR). This combines power, significance and effect size. Constraints are what defines strong evidence, the chance of finding this if H1 is true, plus the risk of misleading strong evidence favouring H1 when H0 is true.

Other constraints are the need for Maturity (follow-up enough to observe late outcomes) and Generalizability (reduced by restrictive eligibility criteria that give more power, but also take longer to recruit). Lastly Money: the budget must be consistent with what the proposed funder is prepared to pay.

In reality sample size for a trial is a compromise chosen from many possible alternatives. It should never be taught as if unthinking application of a mathematical formula will suffice.

